# Development and Characterization of Antimicrobial Chitosan/Polyethylene Oxide/Bacterial Cellulose Nanofibers

**DOI:** 10.3390/polym17050693

**Published:** 2025-03-05

**Authors:** Fatma Sude Cetin, Tubanur Avci, Emre Uygur, Elif Ilhan, Elif Kaya, Gulgun Bosgelmez Tinaz, Liviu Duta, Canan Dogan, Oguzhan Gunduz

**Affiliations:** 1 Center for Nanotechnology and Biomaterials Application and Research (NBUAM), Marmara University, Istanbul 34722, Turkey; sudegoktug@hotmail.com (F.S.C.); avcitubanur@gmail.com (T.A.); emreygr@gmail.com (E.U.); eliffguven@gmail.com (E.I.); oguzhan@marmara.edu.tr (O.G.); 2 Department of Metallurgical and Materials Engineering, Faculty of Technology, Marmara University, Istanbul 34722, Turkey; 3 Department of Biochemistry, Health Sciences Institute, Marmara University, Istanbul 34854, Turkey; ekaya14@marun.edu.tr; 4 Department of Basic Pharmaceutical Sciences, Faculty of Pharmacy, Marmara University, Istanbul 34668, Turkey; gulgun.tinaz@marmara.edu.tr; 5 National Institute for Laser, Plasma and Radiation Physics, 409 Atomistilor Street, 077125 Magurele, Romania

**Keywords:** bacterial cellulose, electrospinning, biomaterial, antimicrobial efficiency, biocompatibility testing

## Abstract

This study introduces novel chitosan (CS) and polyethylene oxide (PEO) copolymers reinforced with bacterial cellulose (BC) to fabricate nanofibers using the electrospinning method. SEM analysis confirmed uniform nanofiber formation, with CS/PEO/BC nanofibers (~240 nm) exhibiting a larger diameter than CS/PEO ones (~190 nm). FTIR spectroscopy confirmed BC integration, while Differential scanning calorimetry analysis indicated minimal impact on glass transition temperature. Notably, as compared to CS/PEO nanofibers, the CS/PEO/BC ones demonstrated superior swelling capacity, accelerated biodegradation, and enhanced mechanical (i.e., tensile) properties, with maximum stress and strain values of ~3.41 MPa and ~0.01% vs. ~2.14 MPa and ~0.01%. Antimicrobial assays confirmed activity against bacterial strains, and biocompatibility tests showed high cell viability at day seven (99.26% for CS/PEO/BC nanofibers). These findings highlight the potential of CS/PEO/BC nanofibers as promising candidates for tissue engineering, offering improved strength, biodegradability, and antimicrobial properties.

## 1. Introduction

A biomaterial is defined as any natural or synthetic substance, or a combination thereof, that can be used for an extended period as a whole or part of a system to treat, augment, or replace any tissue, organ, or function of the body [1]. The utilization of biomaterials is crucial due to their biocompatibility, ability to support tissue repair, and potential to enhance the efficacy of medical treatments.

Biomaterials have a wide range of applications across various medical fields. In orthopedics and traumatology, biomaterials are employed in bone and joint prosthetics, as well as in screws and nails designed to restore skeletal structures. In dentistry, they are utilized in dental implants, filling materials, and protective coatings. Within the cardiovascular system, biomaterials are integral components of stents and artificial heart valves, used to manage vascular disorders and heart diseases. For skin and soft tissue applications, they serve as wound dressings, particularly in the treatment of burns. Additionally, they play an essential role in ophthalmology, especially in the development of contact lenses and corneal implants. These diverse applications underscore the versatility and critical importance of biomaterials in modern medicine [2,3,4].

Several key parameters are essential in the development of biomaterials, including biocompatibility, biodegradability, mechanical strength, biomimetic properties, and machinability. Biocompatibility ensures that the material does not provoke an adverse immune response from body tissues and remains non-toxic [5]. Biodegradability refers to the material’s capacity to degrade or decompose naturally within the biological environment over time [6], minimizing long-term residue. Mechanical strength is essential to meet the structural and mechanical demands specific to the tissue or organ being treated [7]. Biomimetic properties enable the material to support cell adhesion, proliferation, and differentiation, thereby mimicking the native characteristics of biological tissues [8]. Lastly, machinability ensures that the material can be readily shaped and processed to meet practical and clinical requirements.

Nanofibers, as a promising biomaterial, have garnered substantial attention in recent years due to their potential applications in tissue engineering. These nanofibers can be fabricated from natural polymers, such as collagen, chitosan (CS), gelatin, casein, and cellulose, as well as synthetic polymers, including polyethylene oxide (PEO), polypropylene, polyvinyl alcohol, polymethyl methacrylate, polylactic acid, and poly(ε-caprolactone) [9,10]. In this study, CS, and bacterial cellulose (BC) were selected as natural polymer sources, while PEO served as the synthetic polymer. CS, derived from the exoskeletons of crustaceans like shrimp and crab, is renowned for its antimicrobial activity against bacteria, fungi, and certain viruses [11,12]. It is also biocompatible, biodegradable, and exhibits low toxicity [13]. BC, a highly pure form of cellulose synthesized by bacteria, is distinguished by its exceptional mechanical strength, high purity, nanofibril structure, and a large surface area, making it a valuable candidate for biomedical applications [14,15]. Additionally, its microporous structure enhances its functionality [16]. PEO, a petroleum-derived synthetic polymer, is water-soluble and biocompatible. Its ability to enhance viscosity [17,18] and its widespread application in medical contexts, including drug delivery and tissue scaffolding, further underscore its significance in this study.

Electrospinning is a versatile technique for fabricating polymer fibers, particularly nanofibers, characterized by sub-micron dimensions and high surface-to-volume ratios. The process involves an electrostatic field to transform a polymer solution into fine fibers, which are subsequently deposited as solid nanostructures on a grounded collector. This method enables precise control over fiber morphology, which is influenced by key parameters such as applied voltage, flow rate, collector distance, and the physical-chemical properties of the polymer solution [19,20,21,22]. Electrospun nanofibers closely mimic the hierarchical structure of the extracellular matrix, making them particularly suitable for various biomedical applications, including tissue engineering, drug delivery systems, and wound dressings. Beyond the biomedical field, electrospinning has garnered attention for its innovative applications in the food and packaging industries. Electrospun fibers enhance the mechanical properties of packaging materials, extend food shelf life, and act as carriers for bioactive compounds such as essential oils and antioxidants. Additionally, they serve as scaffolds for bacterial cultures during fermentation processes and can be integrated with pH-sensitive sensors for smart packaging solutions [21,23].

In this study, nanofibers combining CS and PEO copolymers with BC were fabricated using electrospinning. Unlike previous studies, this approach employs a distinctive combination of materials that enhances processability and structural integrity while leveraging the synergistic effects of BC, CS, and PEO. The integration of these components significantly improves the mechanical properties, antimicrobial activity, and biocompatibility of the resulting nanofibers, offering a novel contribution to the field. By addressing existing challenges in nanofiber production, this study establishes a foundation for the development of eco-friendly, functional biomaterials tailored for advanced biomedical applications.

## 2. Materials and Methods

### 2.1. Materials

PEO (M_w_ = 6000 g/mol) and CS (M_w_ = 100.00 g/mol) were procured from Sigma Aldrich (Saint Louis, MO, USA). Glucose (M_w_ = 180.16 g/mol), citric acid (M_w_ = 192.12 g/mol), and di-sodium hydrogen phosphate (M_w_ = 141.96 g/mol) were purchased from Merck (Darmstadt, Germany). Casein peptone Type I and yeast extract were obtained from Neogen (Lexington, KY, USA), while glacial acetic acid (AA, M_w_ = 60.05 g/mol, ≥100%), dimethylacetamide (DMAc, M_w_ = 87.12 g/mol, ≥99.0%), and lithium chloride (LiCl, M_w_ = 42.39 g/mol) were supplied by ISOLAB (Windsor, ON, Canada).

### 2.2. Bacterial Cellulose Production

*Gluconacetobacter xylinus* (ATCC 23767, Virginia, USA) was used for the production of BC pellicles. The bacteria were cultured in Hestrin and Schramm (HS) medium, composed of 2% (*w*/*v*) glucose, 0.5% (*w*/*v*) yeast extract, 0.5% (*w*/*v*) peptone, 0.27% (*w*/*v*) di-sodium hydrogen phosphate, and 0.115% (*w*/*v*) citric acid per liter of distilled water. The pH of the medium was adjusted to 5.5 with AA, as this value was demonstrated to be optimal for cellulose production [24]. The medium was sterilized by autoclaving at 121 °C for 20 min and cooled to room temperature (RT) before use.

Cells were initially precultured in test tubes and then inoculated into a 100 mL Erlenmeyer flask containing 40 mL of HS medium. The flask was incubated statically at RT for 3 days. Upon the appearance of a thin cellulose layer on the surface, 1% of the cells and medium were transferred to a new container and subsequently to Petri dishes, where they were incubated at RT for 7 days.

To purify the cellulose pellicles, they were immersed in a 0.1 M sodium hydroxide solution for 24 h at RT to remove cells and residual components of the culture medium. The pellicles were then neutralized overnight by washing with deionized water. For sterilization, the pellicles were autoclaved again and stored at −20 °C until further use. Before use, frozen pellicles were thawed and prepared by freeze-drying.

**Figure 1:**
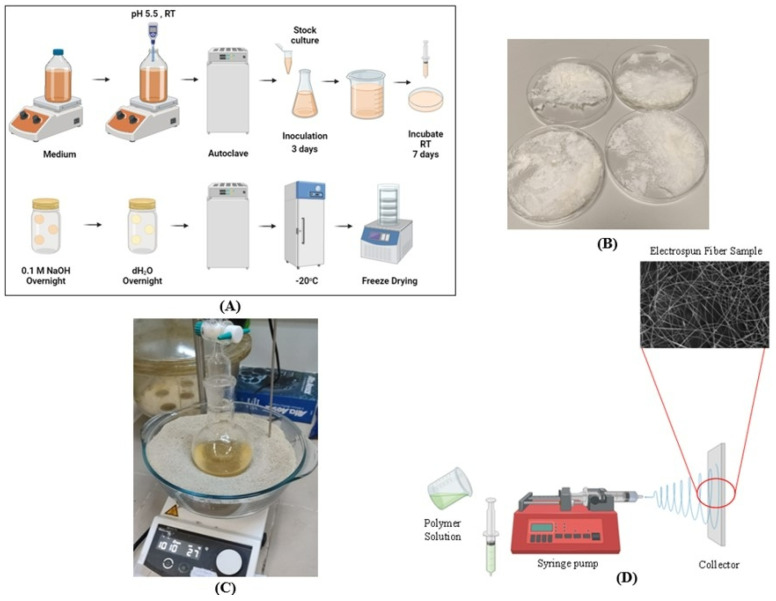
(**A**) Process of bacterial cellulose production, (**B**) Freeze-dried bacterial cellulose, (**C**) Bacterial cellulose dissolved using a sand bath, (**D**) Schematic representation of the electrospinning process.

As illustrated in Figure 1, the BC production process involves culturing and purification steps, culminating in the electrospinning setup, where the prepared BC is utilized for nanofiber fabrication.

### 2.3. Dissolution and Acetylation of Bacterial Cellulose

A 100:1 (*v*/*v*) mixture of DMAc and BC was stirred and heated at 170 °C for 1 h using a sand bath in a condenser system. Subsequently, LiCl (0.4%) was added to the mixture, which was then stirred at 100 °C for an additional hour. The mixture was further stirred at RT for 24 h, ultimately resulting in the formation of a viscous substance.

### 2.4. Electrospinning Process

CS was prepared by dissolving a 10% (*v*/*v*) AA-water solution at a concentration of 3 wt.%. BC was dissolved at concentrations of 5, 7, and 10 wt.% in DMAc, while PEO was dissolved at 2 and 5 wt.% in distilled water, each at RT with continuous stirring for 4 h. Subsequently, 3% CS, 5% BC, and 5% PEO solutions were mixed in a 1:1:1 ratio and stirred overnight at RT.

The prepared solutions were immediately electrospun using a system consisting of a 10 mL syringe with an 18-gauge needle (inner diameter: 0.84 mm), a ground electrode (21.5 cm diameter stainless steel sheet mounted on a rotating drum with variable speed), and a high-voltage source. The electrospinning process was conducted at a voltage of 25 kV, a working distance of 15 cm, and a solution flow rate of 0.5–0.7 mL/h. All electrospinning procedures were performed at RT, and the resulting nanofibers were stored at RT for subsequent characterization tests.

### 2.5. Scanning Electron Microscopy (SEM)

The morphological microstructure of the nanofibers was analyzed by SEM (ZEISS, Jena, Germany). To prevent electrostatic charging and enhance image resolution, the nanofiber samples were coated with a gold layer for 120 s using a sputtering technique. The diameters of the nanofibers were measured using ImageJ software (v1.47, NIH, Bethesda, MD, USA) for precise analysis.

### 2.6. Fourier Transform Infrared (FTIR) Spectroscopy

FTIR analysis was conducted using a FT/IR-4700 spectrometer (JASCO Corporation, Tokyo, Japan) to qualitatively identify the components of the nanofiber samples. The spectra were recorded in the range of 4000–400 cm^−1^, with 32 scans performed at a resolution of 4 cm^−1^.

### 2.7. Differential Scanning Calorimetry (DSC)

A DSC-60 Plus instrument (Shimadzu Corporation, Kyoto, Japan) was used to investigate the thermal properties of the nanofibers. Approximately 2–3 mg of nanofiber samples were placed in aluminum pans and positioned in the DSC sample holder. The thermal analysis was conducted by heating the samples from 25 °C to 300 °C at a rate of 10 °C/min.

### 2.8. Swelling-Degradation Test

The swelling-degradation test is commonly conducted to evaluate the response of a material when exposed to a specific liquid or medium. To assess the swelling and degradation properties of the produced nanofibers, the initial dry mass (*W_d_*, Equation (1)) of each sample was recorded. The nanofibers were then immersed in 1 mL of phosphate-buffered saline (PBS) at pH 7.4 for the swelling test. The samples were incubated at RT in a thermal shaker for 24 h.

At 24 h intervals, the samples were removed from the solution, and excess liquid on the surface was gently blotted using filter paper. The swollen mass (*W_w_*) of each sample was then measured (Equation (1)). This procedure was repeated at regular intervals for up to 336 h. The swelling ratio was calculated using the following formula [25]:

(1) $$S=\frac{W_{w}-W_{d}}{W_{d}}\times 100$$

For the degradation test, the initial mass (*W*_0_, Equation (2)) of each sample was measured. The samples were then immersed in 1 mL of PBS at pH 7.4 and incubated in a thermal shaker at RT for 24 h. After incubation, the samples were removed from the PBS, dried in an oven at 37 °C for 24 h, and their dry mass (*W_t_*) was recorded (Equation (2)). This process was repeated at 24 h intervals for up to 168 h. The degradation rate was calculated using the following formula [25]:

(2) $$D=\frac{W_{0}-W_{t}}{W_{0}}\times 100$$

### 2.9. Tensile Test

To evaluate the mechanical properties of the nanofiber patches, tensile testing was performed using a Shimadzu EZ-LX device (Shimadzu Corporation, Kyoto, Japan). The nanofiber patches were prepared as rectangular specimens measuring 10 mm × 50 mm. Prior to testing, the thickness of each patch was measured using a digital micrometer (Mitutoyo MTI Co., Dusseldorf, Germany). The tensile test was conducted at a speed of 5 mm/min with an applied force of 0.1 N. Each measurement was performed in triplicate for each group to ensure accuracy and reproducibility.

### 2.10. Antimicrobial Testing

The microorganisms *E. coli* (ATCC 25922) and *S. aureus* (ATCC 23235) were inoculated onto 5% sheep blood Columbia Agar (COS; Biomerieux, Isère, France) and McConkey Agar (MCK; Biomerieux, Isère, France) using dilution seeding methods. The inoculated plates were incubated overnight at 35 °C. For further testing on Mueller Hinton Broth medium (MHB; Biomerieux, Isère, France), bacterial suspensions were prepared with a turbidity of 0.5 McFarland (1–5 × 10^8^ CFU/mL) and spread onto Mueller Hinton E. Agar (MHE; Biomerieux, Isère, France).

For these tests, the disk diffusion method was employed, Thus, small circular disks (of ~5–6 mm) were cut from each scaffold, sterilized under UV light for 10 min, and placed in the Petri dish. As recommended by the protocol [26], a distance of 12–20 mm was maintained between the samples and from the edge of the Petri dish to prevent any potential interference of the samples. Inhibition zones were determined by measuring the areas where there was no microbial growth.

### 2.11. Cell Culture Assay

Human dermal fibroblast (HDF) cell lines (ATCC-PCS-201-012) were grown in a 5% CO_2_ incubator at 37 °C (ThermoFisher, Waltham, MA, USA) using Dulbecco’s Modified Eagle Medium (DMEM) (Gibco, Carlsbad, CA, USA). When the cells reached ~80% confluency, they were detached using trypsin/EDTA (Gibco, Carlsbad, CA, USA) and harvested. The detached cells were centrifuged at 2500 rpm for 5 min to form a pellet, which was subsequently lysed in an appropriate volume of DMEM. The total number of cells was quantified using a cell counting device (Anvajo Science, Dresden, Germany).

To assess cell viability, circular disks with a 6 mm diameter were cut from the nanofibers and sterilized under UV light for 2 h. Following sterilization, the disks were rinsed with PBS and transferred into a 96-well plate. Each well was subsequently inoculated with DMEM supplemented with 10% fetal bovine serum (FBS) and 1% penicillin/streptomycin, followed by a 24 h incubation period to stabilize the system.

HDFs were seeded onto the sterilized nanofibers at an initial density of 5 × 10^3^ cells per well. As a control, an equivalent number of cells were seeded directly onto empty wells (2D control). Both the cell-seeded nanofibers and the control samples were incubated at 37 °C with 5% CO_2_ for one week.

Cell proliferation was assessed using the 3-(4, 5-dimethylthiazolyl-2)-2, 5-diphenyltetrazolium bromide (MTT) assay (ElabScience, Houston, TX, USA). At 1, 3, and 7 days post-seeding, MTT solution was added to the wells and incubated for 3 h at 37 °C. Dimethyl sulfoxide was added to solubilize the formazan crystals formed by metabolically active cells. The absorbance of the resultant formazan product was measured at 570 nm using a microplate reader (Agilent BioTek, Epoch, Santa Clara, CA, USA). All experiments were conducted in triplicate to ensure reproducibility and statistical robustness.

### 2.12. Statistical Analysis

Statistical significance was evaluated using one-way ANOVA, followed by Turkey’s post hoc test. A significance level of *p* < 0.05 was considered, with data labeled as follows: (*) for *p* < 0.05, (**) for *p* < 0.01, (***) for *p* < 0.001. Results are expressed as mean ± standard deviation (SD). ImageJ software (v1.47, NIH, Bethesda, MD, USA) was used to determine the nanofiber diameter from the SEM measurements.

## 3. Results and Discussion

### 3.1. SEM Investigation

**Figure 2:**
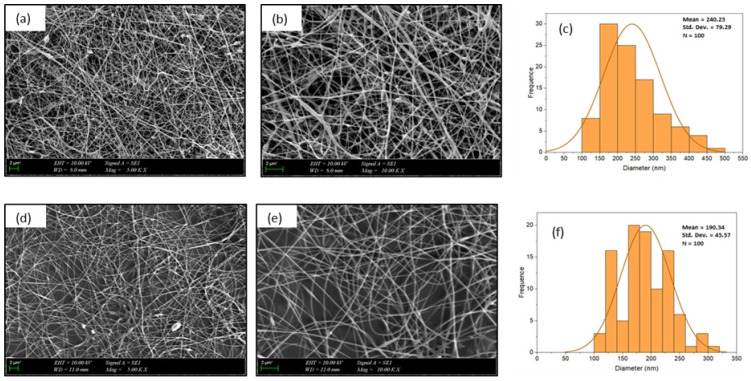
SEM images and diameter distributions of CS/PEO/BC and CS/PEO nanofibers: (**a**,**b**) CS/PEO/BC nanofibers at ×5000 and ×10,000 magnifications, respectively; (**c**) Diameter distribution of CS/PEO/BC nanofibers; (**d**,**e**) CS/PEO nanofibers at ×5000 and ×10,000 magnifications, respectively; (**f**) Diameter distribution of CS/PEO nanofibers.

The SEM images and corresponding histograms of fiber diameter distributions for CS/PEO/BC and CS/PEO nanofibers are shown in Figure 2. The SEM images clearly depict well-defined fibers with a uniform diameter distribution, homogeneous structure, and smooth morphology in both nanofiber architectures.

The average diameter of CS/PEO nanofibers was measured at 190 ± 44 nm, while CS/PEO/BC nanofibers exhibited a larger diameter of 240 ± 79 nm. As illustrated in the histogram graphs, the incorporation of BC led to a noticeable increase in fiber diameter. This effect can be attributed to the high crystallinity and fibrillar network of BC, which contribute to nanofiber diameter expansion.

The presence of BC enhances the viscosity of the polymer solution, thereby facilitating the formation of thicker fibers. Additionally, BC’s high surface area and water retention capacity promote the development of a more organized fiber structure. These structural characteristics enable BC to function as a reinforcing component within the polymer matrix, ultimately resulting in an increased fiber diameter [27].

### 3.2. FTIR Spectroscopy Analysis

**Figure 3:**
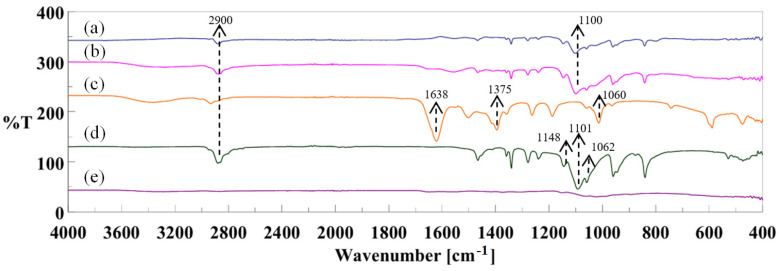
FTIR spectra of: (a) CS/PEO/BC nanofibers, (b) CS/PEO nanofibers, (c) BC, (d) PEO, and (e) CS.

FTIR spectroscopy confirmed the chemical composition of the CS/PEO/BC nanofiber structure, as demonstrated by the spectra presented in Figure 3. The spectra distinctly exhibit the characteristic absorption bands of BC, CS, and PEO, validating their presence within the nanofiber matrix.

A prominent aliphatic C-H stretching vibration peak observed around 2900 cm^−1^ is particularly noteworthy, as it corresponds to the C–H stretching vibrations in both PEO and cellulose, confirming the presence of PEO [28]. This observation is further supported by the sharp peak at approximately 1100 cm^−1^, attributed to the stretching vibrations of characteristic C–O–C ether bonds in the CS/PEO/BC composite.

In pristine BC, distinct absorption bands were identified at 1638 cm^−1^ and 1375 cm^−1^, corresponding to carbonyl groups and CH_2_ bending vibrations, respectively. Additionally, a broad peak at approximately 1060 cm^−1^, associated with glycosidic bonds, was observed [29].

For CS, characteristic absorption peaks were identified at 1680 cm^−1^ and 1538 cm^−1^, corresponding to the carbonyl group (C=O–NHR) and amine (–NH_2_) groups, respectively. Additionally, bands associated with the amide I, amide II, and amide III regions further highlight the structural features of CS.

The presence of PEO was confirmed by the characteristic C–O–C stretching vibrations observed at 1148, 1101, and 1062 cm^−1^, indicative of its polyether chain structure [30]. The incorporation of BC was confirmed by the appearance of characteristic peaks, supporting the formation of a new composite material.

In conclusion, the FTIR spectrum of the CS/PEO/BC composite exhibits the characteristic vibration bands of all three components, confirming the successful integration of their chemical structures.

### 3.3. DSC Analysis

**Figure 4:**
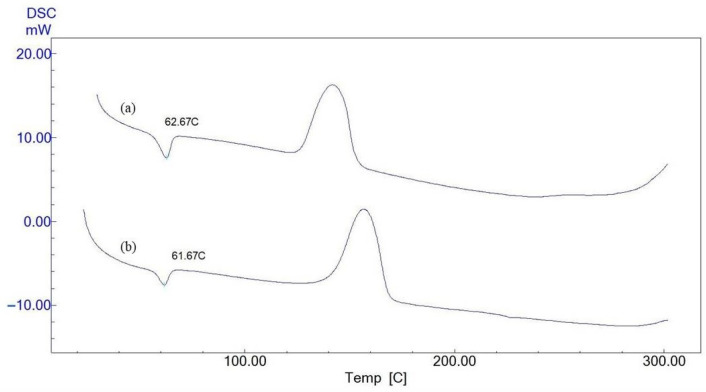
Differential scanning calorimetry curves of: (a) CS/PEO/BC nanofibers, and (b) CS/PEO nanofibers.

The DSC curves of CS/PEO/BC and CS/PEO nanofibers are presented in Figure 4. The glass transition temperatures (*T_g_*) of both nanofibers are remarkably similar, with CS/PEO/BC nanofibers exhibiting a *T_g_* of 62.67 °C, while CS/PEO nanofibers show a *T_g_* of 61.67 °C.

This small difference in *T_g_* indicates that the thermal properties of both compositions are highly comparable. The incorporation of BC resulted in only a 1 °C increase in the glass transition temperature, suggesting that BC has a minimal effect on the glass transition behavior when integrated into the polymer matrix.

The *T_g_* of BC-incorporated fibers is significantly higher than that of native BC (13.94 °C) and NaOH-treated BC membranes (41.41 °C), as reported by George et al. [31]. This elevated *T_g_* reduces material aging at storage temperatures, thereby enhancing the longevity and stability of the composite material [32].

### 3.4. Swelling-Degradation Tests

**Figure 5:**
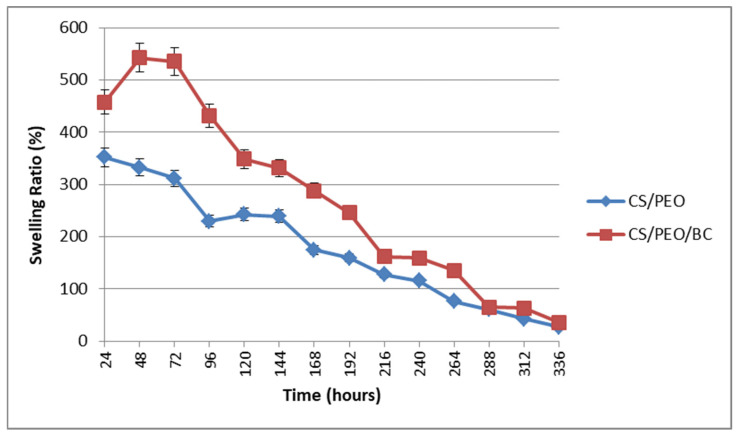
Swelling behavior of nanofiber samples.

The swelling ratio for both investigated group samples exhibited high values within the first 24 h, followed by a slight increase in the CS/PEO/BC group between 48 and 72 h. Over time, a gradual decrease in the swelling ratio was observed in both groups. The reason the swelling rate decreases over time is that the material reaches water saturation and enters the structural stabilization process. Initially, the polymer network structure expands by rapidly absorbing water, but over time, the water uptake is balanced and the polymer chains enter the relaxation process by rearranging. In addition, polymer degradation processes or water evaporation may also contribute to this decrease in swelling ratio [33]. However, the CS/PEO/BC group consistently demonstrates a higher swelling capacity compared to the CS/PEO group, as illustrated in Figure 5.

**Figure 6:**
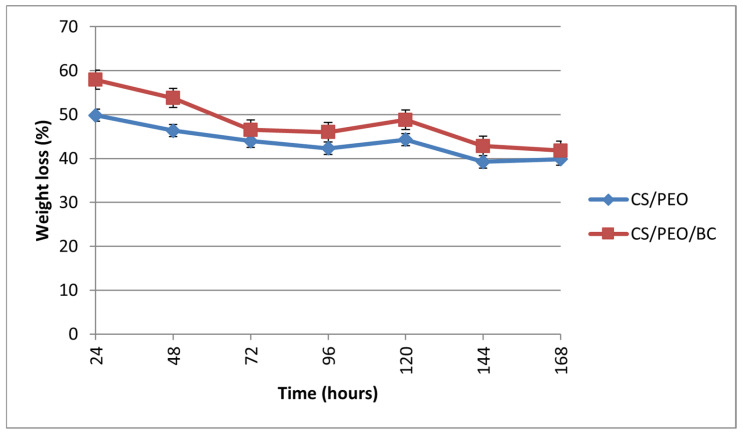
Degradation behavior of nanofiber samples.

The incorporation of BC appears to enhance the swelling ratio and strengthen the material’s interaction with water, likely due to an increase in hydrophilicity [34]. Over time, both groups exhibit mass loss, with the CS/PEO/BC group generally experiencing a slightly greater mass loss compared to the CS/PEO group (Figure 6).

Mass loss occurs most rapidly within the first 24–48 h, after which it stabilizes at a more constant rate. The higher mass loss observed in the CS/PEO/BC group may be attributed to the enhanced biodegradation rate introduced by BC incorporation. This characteristic could be advantageous for biomedical applications, particularly in controlled drug release systems [35].

### 3.5. Tensile Tests

The mechanical behavior of nanofibrous meshes, including tensile strength and strain at break, has been comprehensively analyzed to assess their applicability across various fields. Key factors such as material composition and intrinsic chemical interactions play a pivotal role in determining these properties, ultimately influencing the durability and performance of the nanofibers.

**Figure 7:**
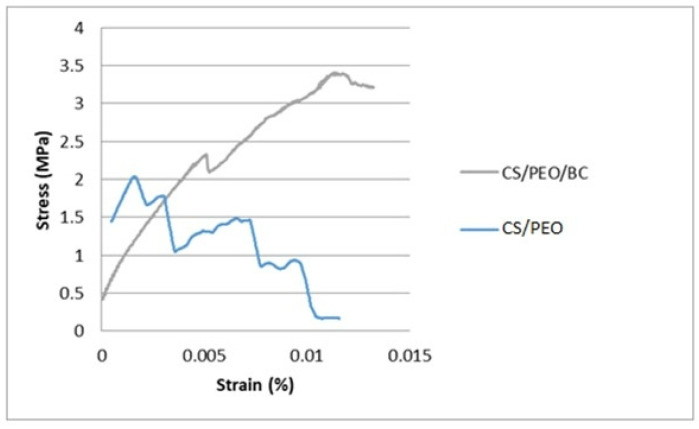
Representation of strain vs. stress in the case of CS/PEO and CS/PEO/BC nanofiber samples.

Tensile testing is particularly significant for biomaterials, as it provides critical insights into their strength and flexibility, which are critical for ensuring functionality and reliability in biomedical applications, such as tissue engineering and drug delivery systems. The tensile testing results for both electrospun nanofiber types are presented in Figure 7.

In this study, the control group of CS/PEO nanofibers exhibited a maximum stress of 2.139 ± 0.532 MPa and a maximum strain of 0.012 ± 0.003%. In a study conducted by Erdem et al., the tensile strength of CS/PEO nanofibers was reported as 4.2 MPa, with a tensile strain of 4.7% [36]. The higher strain values reported in their study can be attributed to the smaller fiber diameters, as the CS/PEO (60/40%) nanofibers had an average diameter of 72 ± 15 nm. In contrast, the CS/PEO nanofibers in our study exhibited and average diameter of 190 ± 44 nm, which likely contributed to the lower strain values observed in our results. Notably, the incorporation of BC into the nanofiber matrix resulted in an increase in both maximum stress (3.407 ± 0.824 MPa) and maximum strain (0.013 ± 0.004%), highlighting its significant impact on enhancing the mechanical properties of the nanofibers.

The superior mechanical performance can be attributed to the high crystallinity of BC and its strong internal and external hydrogen bonding, which contribute to an increased fiber diameter and the formation of a robust internal network. These structural characteristics enhance the mechanical durability and resilience of the nanofibers [23,37].

### 3.6. Antimicrobial Activity

**Figure 8:**
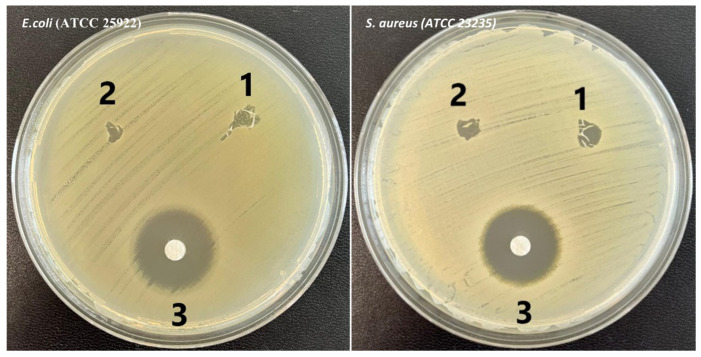
Antimicrobial effects of (**1**) CS/PEO nanofibers, (**2**) CS/PEO/BC nanofibers, and (**3**) Control (Gentamicin) against *E. coli* and *S. aureus*.

The antibacterial effects of the nanofibers against both microorganisms are illustrated in Figure 8, with the corresponding values summarized in Table 1.

The diameters of the inhibition zones for all nanofiber samples against both microorganisms were measured according to the CLSI guidelines, which define the zone boundary—including the disk diameter—as a distinct, visible, non-growing area detectable by the naked eye. The results confirmed the antimicrobial activity of both investigates samples, with CS/PEO nanofibers exhibiting slightly higher efficacy compared to CS/PEO/BC ones. Specifically, the maximum inhibition zone against *S. aureus* was 7 mm for CS/PEO nanofibers and 6 mm for CS/PEO/BC ones. Similarly, the inhibition zones against *E. coli* were 6 mm and 4 mm for CS/PEO and CS/PEO/BC nanofibers, respectively.

Possible explanations for the irregular inhibition zones observed in samples 1 and 2 include: (i) uneven diffusion of the tested compounds, potentially caused by differences in their solubility or viscosity, which may have led to an asymmetric distribution within the agar medium; and/or (ii) interaction with the medium, which could have influenced the diffusion dynamics of the tested compounds, resulting in a non-uniform distribution. It should be emphasized that the control zone (i.e., zone 3) was created using a standard antibiotic disk, which is known to diffuse uniformly within the agar medium, resulting in a well-defined, circular inhibition zone. In contrast, both CS/PEO and CS/PEO/BC nanofibers, unlike the control disk, possess a very fine structure, making it challenging to cut them into a smooth, circular shape. Consequently, the inhibition zones they formed were irregular. Furthermore, the differences observed in samples 1 and 2 are likely attributed to their distinct chemical or physical properties, which may have influenced their diffusion behavior compared to the control.

The literature highlights the broad-spectrum antimicrobial properties of CS, which can disrupt bacterial cell walls and compromise their structural integrity. This characteristic makes CS highly promising for applications in food safety and agriculture.

Numerous studies have demonstrated that CS-based biomaterials effectively inhibit microbial growth, exhibiting antimicrobial efficacy against both Gram-positive and Gram-negative bacteria. This effectiveness has been validated across various pH conditions and concentrations [38,39], further supporting the potential of CS-based materials in antimicrobial applications.

In contrast, the high hydrophilicity of BC can hinder the penetration of antimicrobial agents, thereby reducing their effectiveness. Additionally, the crystalline structure of BC may restrict the diffusion of antibacterial agents within the matrix, further contributing to its relatively lower antimicrobial activity.

These factors provide valuable insights into the subtle differences observed in the antimicrobial effects between CS and BC [40,41].

### 3.7. Cell Viability Tests

**Figure 9:**
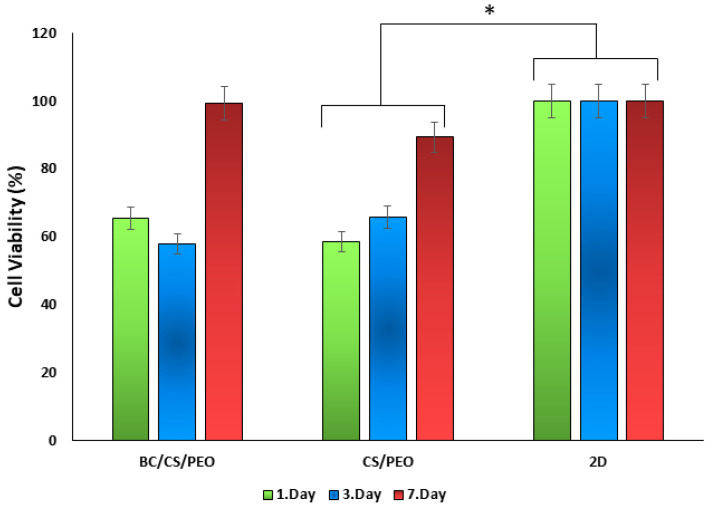
Cell viability of the human fibroblast cell line cultured on nanofiber samples over the designated time periods (* *p* < 0.05).

The proliferation of human fibroblast cell lines cultured on the fabricated material was assessed at 1, 4, and 7 days post-seeding using the MTT assay (Figure 9). By the seventh day, the cell viability of both CS/PEO/BC and CS/PEO nanofibers was determined to be 99.26% and 89.32%, respectively.

The seven-day MTT study demonstrated that cell viability in all groups exceeded 70% compared to the control group. Across both groups, cell viability presented reduced values on days one and three but increased significantly by day seven. This trend is consistent with the typical cellular response to a new culture environment, where cells experience initial stress during the adaptation phase, temporarily reducing viability. Over time, cells acclimate to their new conditions and begin to proliferate, leading to a notable recovery and increased viability [42]. The observed decrease in cell viability on days one and three for both scaffolds can be attributed to this initial adaptation phase, followed by a recovery to satisfactory viability levels by day seven.

The CS used in nanofibers is a polymer with well-documented biocompatibility, as demonstrated in numerous previous studies. Its high biocompatibility is attributed to its structural and functional resemblance to glycosaminoglycans, which are integral components of the extracellular matrix in the human body [43,44].

The elevated biocompatibility values observed on day seven in both nanofiber groups can be primarily attributed to the CS content within the nanofiber composition. On day three, a slight decrease in BC/CS/PEO nanofibers is observed compared to CS/PEO nanofibers. Although BC exhibits excellent biocompatibility and water retention capacity, it may somewhat hinder cell adhesion. Additionally, its dense fibrous structure could limit cell penetration and spreading [45]. The decrease in cell viability on day three may be attributed to this factor. However, cells gradually adapt to their environment from day one to day seven. Although BC slightly inhibits cell viability, it exhibits an exceptional capacity for water absorption and retention, creating a moist microenvironment that supports cell viability, proliferation, and differentiation [46]. The slightly higher cell viability observed in the CS/PEO/BC scaffold compared to the CS/PEO scaffold on day seven is likely attributed to the enhanced biocompatibility conferred by BC.

## 4. Conclusions

In this study, nanofibers were successfully developed by integrating bacterial cellulose (BC), chitosan (CS), and polyethylene oxide (PEO) via electrospinning. The CS/PEO nanofibers exhibited a tensile strength of ~2.14 MPa and an elongation of ~0.01%. With the incorporation of BC, the tensile strength of CS/PEO/BC nanofibers increased by ~59%, reaching ~3.4 MPa, while maintaining a similar elongation (~0.01%). Additionally, the composite nanofibers demonstrated a water absorption capacity exceeding 200% within 24 h, highlighting their potential for tissue engineering and wound-healing applications. Cell viability assays indicated a 99.26% viability rate on day seven, confirming the excellent biocompatibility of CS/PEO/BC nanofibers and their ability to support cell proliferation. These findings align with previous reports demonstrating that BC incorporation significantly enhances the mechanical strength, water retention capacity, and biocompatibility of nanofibrous scaffolds.

In conclusion, CS/PEO/BC nanofibers, with their improved mechanical properties, superior water retention capacity, and excellent biocompatibility, hold significant potential for biomedical applications, particularly in tissue engineering.

## Figures and Tables

**Table 1:** Antimicrobial activity of the nanofibers, presented as the mean inhibition zone diameters (mm) against *E. coli* and *S. aureus*.

Type of Bacteria	Zone of Inhibition—Mean Diameter [mm]		
	CS/PEO	CS/PEO/BC	Control (Gentamicin/10 µg)
*E. coli*	6	4	20
*S. aureus*	7	6	19

## Data Availability

The original contributions presented in this study are included in the article. Further inquiries can be directed to the corresponding authors.

## List of Abbreviations

AA	acetic acid
CS	Chitosan
CS/PEO/BC	Chitosan and polyethylene oxide combined with bacterial cellulose
BC	bacterial cellulose
CFU	colony forming units
DMAc	dimethylacetamide
DMEM	Dulbecco’s Modified Eagle Medium
DSC	Differential scanning calorimetry
FBS	fetal bovine serum
FTIR	Fourier Transform Infrared spectroscopy
HDF	Human dermal fibroblast
HS	Hestrin and Schramm medium
LiCl	lithium chloride
MHB	Mueller Hinton Broth medium
MHE	Mueller Hinton E. agar
MTT	3-(4, 5-dimethylthiazolyl-2)-2, 5-diphenyl-238 tetrazolium bromide
NaOH	sodium hydroxide
PBS	phosphate-buffered saline
PEO	Polyethylene oxide
RT	room temperature
SEM	scanning electron microscopy
SD	standard deviation
*T_g_*	glass transition temperature
*W*_0_	initial mass
*W_d_*	initial dry mass
*W_t_*	dry mass
*W_w_*	swollen mass
